# Reported firearm access among patients admitted to a dual diagnosis medically-assisted withdrawal unit over five years

**DOI:** 10.1016/j.dadr.2022.100034

**Published:** 2022-03-02

**Authors:** Jeremy Weleff, Robert S. Butler, David Streem, Brian S. Barnett

**Affiliations:** a Department of Psychiatry and Psychology, Center for Behavioral Health, Neurological Institute, Cleveland Clinic, Cleveland, OH, USA; b Quantitative Health Sciences, Cleveland Clinic, Cleveland, OH, USA; c Cleveland Clinic Lerner College of Medicine at Case Western Reserve University, EC-10 Cleveland Clinic, 9501 Euclid Ave, Cleveland, OH, USA

**Keywords:** Firearms, Guns, Suicide, Homicide, Alcohol use disorder, Opioid use disorder, Risk assessment, Detox, withdrawal

## Abstract

**Background:**

Up to one-third of firearm-related suicides were carried out by individuals who had consumed alcohol shortly before their death. Despite the critical role of firearm access screening in suicide risk assessment, few studies have examined firearm access among patients with substance use disorders. This study examines the rates of firearm access among those admitted to a co-occurring diagnosis unit over a five year period.

**Methods:**

All patients admitted to a co-occurring disorders inpatient unit from 2014 to mid-2020 were included. An analysis contrasting the differences among patients reporting firearms was performed. A multivariable logistic regression model using factors from initial admission were chosen based on clinical relevance, past firearms research, and statistical significance on bivariate analysis was used.

**Results:**

Over the study period there were 7332 admissions representing 4055 patients. Documentation of firearm access was completed in 83.6% of admissions. Firearm access was reported in 9.4% of admissions. Patients reporting firearm access were more likely to report never having suicidal ideation (*p* = 0.001), be married (*p* = <0.001), and report no past history of suicide attempts (*p* = <0.001). The full logistic regression model revealed that being married (OR: 2.29 and *p* < 0.0001) and employed (OR: 1.51 and *p* = 0.024) were factors associated with firearms access.

**Conclusions:**

This is one of the largest reports assessing factors associated with firearm access among those admitted to a co-occurring disorders unit. Firearm access rates in this population appear lower than rates in the general population. The roles employment and marital status play in firearm access deserve future attention.

## Highlights

• Limited data exist on firearm access among those with substance use disorders.
• Firearm screening allows for higher rates of completed documentation.
• Marital status and employment appear to be related to firearm access.
• Those with substance use disorders report much lower rates of firearm access than the general population.

## 1 Introduction

There were 39,707 deaths related to firearms in the United States in 2019 (Centers for Disease Control and Prevention; National Centers for Injury Prevention and Control., 2021). Of those deaths, 60% were suicides, 37% were homicides, and the remaining 3% were unintentional, resulting from legal intervention, mass shooting, or undetermined causes. In addition to firearm deaths, there are approximately 115,000 non-fatal firearm related injuries in the United States annually. Firearms are implicated in approximately half of completed suicides and are one of the most lethal means of suicide, with nearly 90% of suicidal acts involving a firearm resulting in death (Conner et al., 2019). Addiction and intoxication are well-established risk factors for both self-directed and interpersonal firearm violence (Wintemute, 2015). For example, up to one-third of firearm-related suicides and homicides were carried out by individuals who had consumed alcohol shortly before their death (Branas et al., 2016).

Despite the critical role of firearm access screening in suicide and violence risk assessment, few studies have examined firearm access and ownership among patients with mental illness or substance use disorders presenting to healthcare facilities for treatment. Existing studies indicate that approximately 11% of people presenting to emergency departments with suicidal ideation or a recent suicide attempt report having access to a firearm (Betz et al., 2018), as well as 9.0–14.6% of patients admitted to inpatient psychiatric facilities (Kolla et al., 2011; McNiel et al., 2007). Patients report being open to firearm safety discussions with their healthcare provider (Hudak et al., 2021), but studies indicate that firearm access is rarely recorded in patients’ charts, complicating public health interventions. For patients presenting to emergency departments with suicidal ideation, rates of documentation of firearm or lethal means access range from 3% to 47% (Betz et al., 2018; Katz et al., 2019; Naganathan and Mueller, 2019). Data on firearm access in patients with substance use disorders are particularly limited, but show cause for concern given the well-established link between many substance use disorders and suicide (Esang and Ahmed, 2018) and violence (Douglas, 2015). Data on patients undergoing detoxification from substances are particularly rare. One study of opioid (*n* = 386) and alcohol users (*n* = 51) admitted for inpatient detoxification in Fall River, Massachusetts, a city of approximately 90,000 people, found higher rates of gun involvement (i.e. keeping gun in the home, carrying gun for protection, gun playing a role in arrests, being wounded by gunshot, etc.) among primary opioid users than among primary alcohol users, with similar rates of gun ownership or access rates (34% and 31%, respectively) (Stein et al., 2018).

Having a more complete understanding of firearm access rates and factors associated with firearm access among individuals with substance use disorders, particularly among individuals with severe disorders requiring detoxification, may prove valuable for improving suicide and violence risk interventions within this vulnerable population. Therefore, we sought to investigate rates of firearm access and assess potential predictors of access in a cohort of patients admitted to a co-occurring disorders inpatient unit in Cleveland, Ohio from 2014 to mid-2020.

## 2 Methods

### 2.1 Study design and population

Medical records for all patients admitted to an urban co-occurring disorders inpatient unit in Cleveland, Ohio from January 1st 2014- May 16, 2020 were included in this retrospective chart review. The unit is primarily for patients requesting medical detoxification and is voluntary in nature. For patients admitted multiple times during the study period, only data from the first admission were used in analyses. Unit intake procedures require that all admitted patients undergo a standardized suicide screening risk assessment that is completed by nurses, resident physicians, or staff physicians and entered directly into intake note in the electronic medical record (EMR). This assessment includes questions about firearm access and other suicide risk factors including current suicidal ideation, past suicide attempts, past suicide planning, history of non-suicidal self-injurious behavior (NSSIB), family history of suicide, and recent inpatient psychiatric treatment. Firearm access was defined by having a positive response to the question, “Any firearms in the home?” Social and demographic information about marital status, ethnicity, gender, age, sexuality, isolated home environment, and dependents is also captured during this interview.

We also extracted primary diagnoses precipitating admission and date/time of admission from the EMR. Time of admission was divided into two 12 h periods designating daytime admission (0800 to 2000) and nighttime admission (2001 to 0759). Patient area of residence was classified as rural, large town, or urban based on zip codes of their home addresses using the Rural-Urban Commuting Area (RUCA) classification from the United States Department of Agriculture. RUCA codes assign census tracts use a value from 1 to 10 that reflects population density and travel/commuting distance to urban centers; urban was defined as RUCA codes 1–3, large town as RUCA codes 4–6, and rural as RUCA codes 7–10.

### 2.2 Ethics approval

This study was approved by the Cleveland Clinic Institutional Review Board (study number 20-182).

### 2.3 Statistical analysis

An analysis contrasting the differences among patients regarding firearms access classification (yes, no, and not assessed) was performed using individual categorical and continuous patient factors. Differences in categorical measures were assessed using either a Chi-square test or Fisher's exact test. Differences in continuous measures were assessed using one-way ANOVA. The reported p-value in the right-hand column in each of the tables in this article is a p-value for the test across the three categories of firearms possession classification. The superscripts within each factor across patient classifications indicate the pairwise comparisons which were statistically significant. A Bonferroni correction was used to adjust for multiple comparisons. In all cases a *p*-value <0.05 was used as the threshold for statistical significance.

We constructed a multivariable logistic regression model using factors chosen based on clinical relevance, results of past firearms research in similar populations and statistical significance on bivariate analysis. The variables of interest included in this multivariable model were gender; marital status; employment status; admission diagnosis; self-reported living in a rural/isolated home environment; living with others in the home; whether the patient had dependents; family history of suicide; history of personal suicide attempts; history of past suicidal intent with a plan; history of past suicidal intent with a plan that specifically did not include a firearm; NSSIB; recently reported psychiatric inpatient treatment; lesbian, gay, bisexual, and transgender (LGBT) identity; daytime vs nighttime admission; and age. The list of possible factors to be included in the multivariable logit model was examined using the methods of Variance Inflation Factors (VIF) and condition indices to check for co-linearity. Factors with a condition index of 10 or greater (ethnicity and RUCA/zipcode classification) were considered to be too highly confounded with other variables in the list and were not included in the list of variables for the multivariable study. This assessment revealed that admission diagnosis could be included in the multivariate analysis and, with the exception of cocaine use disorder and polysubstance use disorder due to their low prevalence, contrasts of admission diagnoses with respect to alcohol use disorder could be estimated. All of the terms with acceptable levels of independence from one another were included in a full logistic regression model. The method of backward elimination regression was used to examine the full model and construct a reduced model consisting of only those terms which remained statistically significant (*P* < 0.05). Just two terms, employment status and marital status, were statistically significant.

## 3 Results

Over the study period there were 7332 admissions to inpatient detoxification representing 4055 unique patients, with a mean (± standard deviation) of 1.8 ± 1.3 (range: 1–11) hospitalizations per patient during the study period. Documentation of completed screening for firearm access was completed in 83.6% of admissions (*n* = 6133, total *n* = 7332). Firearm access was reported in 9.4% (*n* = 691) of all admissions and for 9.9% (*n* = 400) of all unique patients. There was no documentation of firearms screening for 16.4% of all admissions (*n* = 1199) and 16.4% of unique patients (*n* = 665).

Results reported hereafter will only include data from the first admission during the study period for each unique patient. 64.0% (*n* = 2595) of patients were male and 78.5% (*n* = 3185) were Caucasian. Patients were primarily single (55.8%, *n* = 1179), unemployed (57.9%, *n* = 1024), residents of urban areas (95.6%, *n* = 3871), and admitted for treatment of alcohol use disorder (77.6%, *n* = 3092). Mean length of stay was 3.5 ± 1.9 days (range: 0–19). 3.1% (*n* = 97) of patients had recently moved a firearm out of their home due to mental health concerns, while 19.2% (*n* = 507) reported a past suicide attempt and 11.9% (*n* = 357) had experienced suicidal ideation with a suicide plan at some point in their lives. 13.3% (*n* = 358) reported a family history of suicide. For further demographic information see Table 1a.

**Table 1a:** Variable summary for unique patient records and associations with firearm access.

		Firearm Access			
Factor	Total (*N* = 4055)	No (*N* = 2990)	Yes (*N* = 400)	Not Assessed (*N* = 665)	*p*-value
Gender*					0.79^c^
Male	2595(64.0)	1909(63.9)	253(63.3)	433(65.1)	
Female	1459(36.0)	1080(36.1)	147(36.8)	232(34.9)	
Ethnicity					0.29^c^
Caucasian	3185(78.5)	2355(78.8)	310(77.5)	520(78.2)	
Black	536(13.2)	383(12.8)	55(13.8)	98(14.7)	
Hispanic	110(2.7)	78(2.6)	10(2.5)	22(3.3)	
Declined	224(5.5)	174(5.8)	25(6.3)	25(3.8)	
Age at hospital admission	45.0 ± 12.6	44.9 ± 12.4	45.7 ± 13.1	45.0 ± 13.0	0.51^a^
Length of stay (LOS)*	3.5 ± 1.9	3.5 ± 1.9	3.5 ± 1.9	3.5 ± 2.2	0.64^a^
Admission diagnosis*					***<0.001^c^***
Alcohol	3092(77.6)	2316(78.3) 2	293(73.8) 1^,^^3^	483(76.3) 2	
Opioid	194(4.9)	148(5.0)	17(4.3)	29(4.6)	
Benzodiazepine	235(5.9)	182(6.2)	24(6.0)	29(4.6)	
Medical Condition	248(6.2)	165(5.6)	28(7.1)	55(8.7)	
Psychiatric disorder	120(3.0)	78(2.6)	28(7.1)	14(2.2)	
Cocaine	7(0.18)	5(0.17)	0(0.0)	2(0.32)	
Polysubstance use	87(2.2)	59(2.0)	7(1.8)	21(3.3)	
Cannabis	3(0.08)	3(0.10)	0(0.0)	0(0.0)	
RUCA/Zip code designation*					0.39^c^
Rural	51(1.3)	37(1.2)	4(1.0)	10(1.5)	
Large Town	127(3.1)	101(3.4)	13(3.3)	13(2.0)	
Urban	3871(95.6)	2848(95.4)	382(95.7)	641(96.5)	
Year of admission					***<0.001^c^***
2014	699(17.2)	547(18.3) 3	67(16.8) 3	85(12.8) 1^,^^2^	
2015	719(17.7)	557(18.6)	59(14.8)	103(15.5)	
2016	709(17.5)	562(18.8)	76(19.0)	71(10.7)	
2017	672(16.6)	478(16.0)	84(21.0)	110(16.5)	
2018	577(14.2)	378(12.6)	55(13.8)	144(21.7)	
2019	518(12.8)	366(12.2)	49(12.3)	103(15.5)	
2020	161(4.0)	102(3.4)	10(2.5)	49(7.4)	
Moved firearm out of home recently*					***<0.001^c^***
No	2996(96.9)	2651(98.8) 2	333(83.9) 1	12(92.3)	
Yes	97(3.1)	32(1.2)	64(16.1)	1(7.7)	
Current suicide plan not including firearm*					0.89^c^
No	3301(99.5)	2867(99.5)	388(99.5)	46(100.0)	
Yes	16(0.48)	14(0.49)	2(0.51)	0(0.0)	
Past history of suicide plans*					***0.001^c^***
No	2642(88.1)	2282(87.3) 2	324(93.9) 1	36(92.3)	
Yes	357(11.9)	333(12.7)	21(6.1)	3(7.7)	
Marital status*					***<0.001^c^***
Single	1719(55.8)	1466(57.4) 2	145(43.2) 1^,^^3^	108(57.4) 2	
Married	653(21.2)	486(19.0) 2	124(36.9) 1^,^^3^	43(22.9) 2	
Divorced	423(13.7)	367(14.4)	34(10.1)	22(11.7)	
Widowed	56(1.8)	48(1.9)	4(1.2)	4(2.1)	
Unknown	228(7.4)	188(7.4)	29(8.6)	11(5.9)	
Family history of suicide*					0.90^c^
No	2334(86.7)	2041(86.8)	267(85.9)	26(86.7)	
Yes	358(13.3)	310(13.2)	44(14.1)	4(13.3)	
History of suicide attempts*					***<0.001^c^***
No	2137(80.8)	1844(79.6) 2	272(90.1) 1	21(87.5)	
Yes	507(19.2)	474(20.4)	30(9.9)	3(12.5)	
Non-suicidal self-injurious behavior (NSSIB)*					0.73^c^
No	2313(93.3)	2028(93.2)	264(94.3)	21(91.3)	
Yes	166(6.7)	148(6.8)	16(5.7)	2(8.7)	
LGBT*					0.12^c^
No	2202(94.5)	1888(94.3)	259(97.0)	55(91.7)	
Yes	127(5.5)	114(5.7)	8(3.0)	5(8.3)	
Recent increase in drug/alcohol use*					0.41^c^
No	278(12.1)	240(12.2)	28(10.4)	10(16.4)	
Yes	2027(87.9)	1735(87.8)	241(89.6)	51(83.6)	
Rural/isolated home environment*					0.59^c^
No	1693(75.2)	1448(74.9)	199(77.7)	46(76.7)	
Yes	557(24.8)	486(25.1)	57(22.3)	14(23.3)	
Family/friends concerned about patient*					0.36^c^
No	109(5.3)	98(5.6)	8(3.4)	3(5.6)	
Yes	1941(94.7)	1660(94.4)	230(96.6)	51(94.4)	
Lives with others*					0.92^c^
No	599(29.7)	513(29.7)	69(29.2)	17(32.1)	
Yes	1417(70.3)	1214(70.3)	167(70.8)	36(67.9)	
Has dependents*					0.18^c^
No	968(49.7)	845(50.5)	99(44.0)	24(48.0)	
Yes	980(50.3)	828(49.5)	126(56.0)	26(52.0)	
Recent psychiatric inpatient treatment*					0.13^c^
No	1663(87.8)	1438(88.3)	186(86.1)	39(79.6)	
Yes	230(12.2)	190(11.7)	30(13.9)	10(20.4)	
Currently employed*					0.093^c^
No	1024(57.9)	889(58.8)	102(50.7)	33(58.9)	
Yes	745(42.1)	623(41.2)	99(49.3)	23(41.1)	
Religious Affiliation*					0.40^c^
No	571(35.8)	490(35.8)	69(38.1)	12(27.3)	
Yes	1024(64.2)	880(64.2)	112(61.9)	32(72.7)	
Day/night admission time					0.44^c^
0800–2000	2987(73.7)	2206(73.8)	302(75.5)	479(72.0)	
2001- 0759	1068(26.3)	784(26.2)	98(24.5)	186(28.0)	

⁎ Data not available for all subjects. Missing values: Gender = 1, Admission diagnosis= 69, Zipcode = 6, Moved firearm out of home recently = 962, Current suicide plan not including firearm = 738, Past history of suicide plans = 1056, Marital Status = 976, Family history of suicide = 1363, History of suicide attempts = 1411, NSSIB = 1576, LGBT = 1726, Recent increase in drug/alcohol use = 1750, Rural/isolated home environment = 1805, Family/friends concerned about patient = 2005, Lives with others = 2039, Has dependents = 2107, Recent psychiatric inpatient treatment = 2162, Currently employed = 2286, Religious Affiliation = 2460, LOS = 9. Statistics presented as Mean ± SD or *N* (column%). *p*-value superscripts refer to method of analysis: *a*=ANOVA, *c*=Pearson's chi-square test.

1 Significantly different from No.

2 Significantly different from Yes.

3 Significantly different from Not Assessed A *p*-value < 0.05 was considered statistically significant. A Bonferroni correction was used for pairwise ad-hoc comparisons.

In the bivariate tests for associations between firearm possession and demographic factors (also summarized in Table 1a) patients possessing a firearm were less likely to: have diagnoses of alcohol use disorder (73.8 vs 78.3%) (*p* = <0.001) and have been admitted in year 2014 when compared to patients who were not assessed for firearms possession (*p* = <0.001). Patients possessing a firearm were more likely to report never having suicidal ideation with a plan (*p* = 0.001), be married (*p* = <0.001), and report no past history of suicide attempts (*p* = <0.001). On multivariable analysis, our full logistic regression model (see Table 2a) revealed that being married (OR: 2.29 and *p* < 0.0001) and employed (OR: 1.51 and *p* = 0.024) were the only assessed factors associated with firearms access. We then developed a reduced multivariable model consisting only of marital status and employment that is summarized within Table 2b. Married patients were 2.54 times more likely to have access to firearms than single patients (*p* < 0.0001) and employed patients were 1.42 times more likely to have firearms access than those unemployed (*p* = 0.044).

**Table 2a:** Full model for firearms access.

			95% WaldConfidenceInterval		
Factor	Contrast	OddsRatio	Lower	Upper	*P*-value
Gender	Male vs Female	0.922	0.64	1.33	0.664
Marital status					
	Married vs Single	2.286	1.532	3.412	**<0.0001**
	Divorced vs Single	0.875	0.486	1.575	0.656
	Widowed vs Single	0.805	0.101	6.427	0.837
	Unknown vs Single	0.961	0.419	2.204	0.924
Currently employed	Yes vs No	1.508	1.054	2.157	**0.024**
Admission diagnosis					
	Opioid Use Disorder vs Alcohol Use Disorder	0.81	0.354	1.853	0.618
	Benzodiazepine/Sedative Use Disorder vs Alcohol Use Disorder	0.997	0.456	2.182	0.995
	Medical Condition vs Alcohol Use Disorder	1.421	0.148	13.66	0.761
	Psychiatric Condition vs Alcohol Use Disorder	0.989	0.111	8.784	0.992
	Cocaine Use Disorder vs Alcohol Use Disorder	<0.001	<0.001	>999.999	0.995
	Polysubstance Use Disorder vs Alcohol Use Disorder	<0.001	<0.001	>999.999	0.979
Rural/isolated home environment	Yes vs No	1.056	0.695	1.602	0.799
Live with others	Yes vs No	0.883	0.569	1.37	0.577
Has dependents	Yes vs No	1.135	0.78	1.651	0.509
Family history of suicide	Yes vs No	1.314	0.75	2.302	0.339
History of suicide attempts	Yes vs No	0.713	0.35	1.454	0.352
Past history of suicide plans	No vs Yes	1.48	0.575	3.812	0.417
Current suicide plan not including firearm	Yes vs No	5.6	0.482	65.113	0.169
Non-suicidal self-injurious behavior (NSSIB)	Yes vs No	1.129	0.504	2.533	0.768
Recent psychiatric inpatient treatment	Yes vs No	1.226	0.685	2.194	0.493
LGBT	Yes vs No	0.623	0.24	1.615	0.33
Day/Night Admission Time	0800-2000 vs 2001- 0759	0.767	0.496	1.185	0.231
Age at hospital admission		1.007	0.992	1.023	0.356

**Table 2b:** Reduced model for firearms access.

			95% WaldConfidenceInterval		
Factor	Contrast	OddsRatio	Lower	Upper	*P*-value
Marital Status					
	Married vs Single	2.543	1.746	3.702	**<0.0001**
	Divorced vs Single	0.957	0.539	1.7	0.881
	Widowed vs Single	0.744	0.095	5.822	0.778
	Unknown vs Single	0.846	0.374	1.915	0.689
Currently employed	Yes vs No	1.420	1.009	2	**0.044**

## 4 Discussion

This study provides a rare look into firearm access rates among patients with substance use disorders, particularly within the inpatient detoxification setting. While a previous study asked patients undergoing detoxification about gun ownership or access within the last year (Stein et al., 2018), we believe ours is the first to assess patients undergoing detoxification for current access to a firearm. We found that employing standard suicidal screening procedures in detoxification settings can yield high rates of completed clinical documentation for firearm access, providing more accurate figures for this important suicide and violence risk factor in this high-risk group. This standardized screening instrument led to documentation rates for firearm access of 83.6% for all admissions. Similar rates have been found in outpatient samples when routine firearm access is assessed (Richards et al., 2021). With most patients entering our detoxification facility via emergency departments, where rates of documentation of firearm or lethal means access range from 3% to 47% (Betz et al., 2018; Katz et al., 2019; Naganathan and Mueller, 2019), it is likely that our facility's approach is capturing information about firearms access that would have otherwise gone either unattained or undocumented for the vast majority of our patients, providing a valuable opportunity for firearm violence prevention in a population at risk for these behaviors.

In recent years, whether clinicians and particularly physicians have the right to ask about firearm access in the home has been a contentious issue, with some commentators arguing that this line of questioning should be left out of the doctor patient relationship (Graham, 2019). This culminated in the “Docs vs Glocks” legal odyssey in which Florida passed a law in 2011 restricting a physician's ability to ask about firearm ownership (Appelbaum, 2017). After a lengthy court battle, that statute was struck down by the U.S. 11th Circuit Court of Appeals in 2017. At this time are no state laws presenting physicians from speaking with patients about firearms in the United States. However, whether physicians should have these conversations remains a subject of intense debate, even within medicine, with 43.6% of readers surveyed by *MD Magazine* in 2016 reporting that physicians do not have a role to play in curbing gun violence compared to 40.3% saying they do (Scott and Kaltwasser, 2016).

Data concerning firearms access and behaviors among those using substances is mixed and there remain many questions about what associations may exist between firearms access and demographic factors, as well as particular substance use disorders and mental illnesses, as previously reviewed (Chen and Wu, 2016). Within this sample, we found no differences in firearm access according to particular admission diagnosis. This is in contrast to a past study of patients undergoing medication-assisted withdrawal which showed higher rates of firearm involvement for those undergoing opioid detoxification compared to those undergoing alcohol detoxification (33.9% vs. 31.4%, respectively) (Stein et al., 2018). The reasons for this remain unclear. Importantly, insurers in Ohio are not required to cover inpatient medically assisted withdrawal from opioids unless patients are concurrently using substances that have life-threatening withdrawal or have other medical conditions that could deteriorate during opioid withdrawal, so our population of patients being treated for opioid withdrawal is not representative of the opioid using population in the surrounding community. There are also notable methodological differences between the aforementioned study and ours, particularly since that study approached firearm involvement very broadly and asked patients about their involvement over the last year and the course of their life while ours assessed only current firearm access. While nationally representative community household survey data suggest that firearm access between those with and without mental illness are similar (Ilgen et al., 2008), two studies of psychiatric inpatients found reported rates of firearm ownership or access of 9.0–14.6% (Kolla et al., 2011; McNiel et al., 2007). The rates of firearm access in this study are similar to the base rates found in psychiatrically hospitalized patients, which are lower than the reported rates in community samples. Interestingly, while there was a statistically significant relationship between firearm access and lower rates of past suicide attempts on bivariate testing, on multivariate logistic regression analysis we found no association between past suicide attempts and firearm access, in contrast to results found in psychiatric inpatients (Kolla et al., 2011), national community samples (Ilgen et al., 2008), and those receiving outpatient care (Richards et al., 2021).

Interestingly, the rates of reported firearm access in our patient population were much lower than that of the general population. While 9.9% of our patients reported firearm access, in a 2019 Gallup poll, 32% of U.S. adults reported personally owning a gun, while 44%, report living in a gun-owning household (Saad, 2020). The rates found in our study were also much lower than state-level rates. In Ohio, the proportion of adults living in a gun-owning household is approximately 36–40% (Schell et al., 2020). Large outpatient samples of those receiving primary care or mental health care have reported rates of firearms access of 20.9% and 15.3%, respectively (Richards et al., 2021). It is unclear why firearm access rates in our population were so low compared to state and national samples of the general population in the United States, though the possibility that patients were not comfortable reporting firearm access to clinicians must be considered, making it likely that our results for firearm access prevalence are an underestimate, perhaps even grossly so. Though we were unable to collect data on income of patients in our sample, it is possible that the low rate of firearm access may be due to low income. Low socioeconomic status is associated with alcohol use disorder (Calling et al., 2019), which was the primary diagnosis for the vast majority of patients treated at our facility, and higher levels of income are associated with firearm ownership and living in a firearm owning household (Parker et al., 2017). Though not thoroughly investigated, there is evidence that individuals with past drug-related charges make up a considerable number of individuals who frequently pawn their goods at pawn shops (Comeau et al., 2011) and guns are a commonly pawned item, drawing the highest price of all items from pawnshops in a Texas study (Carter and Skiba, 2012). An important line of research in the future will be asking those with substance use disorders about past firearms ownership and, determining whether their substance use disorder played a role in selling a firearm versus selling or giving it away due to concerns about their mental health. Other potential explanatory factors include the possibility that some patients may have felony convictions related to possession or distribution of illicit substances and others may have been involuntary committed for inpatient psychiatric care, both of which lead to restrictions on firearm ownership in Ohio (National Conference of State Legislatures, 2021; Ohio Revised Code, 2014) .

We found that being married was associated with firearms access, in line with observations that gun owners and people living in gun owning households in the U.S. are more likely to be married (Saad, 2020). The reasons for this association are not clear. In 2019, personal/safety and protection were the most commonly cited reasons for gun ownership, reported by 63% of gun owners in the U.S. (Gallup, 2022). One possibility is that this motivating factor may be stronger for married individuals, though we did not find associations between firearm access and dependents, making that less likely. Our finding of an association between marriage and firearm access in this population being primarily treated for alcohol use disorder is important since alcohol use is associated with intimate partner violence in general (Foran and O'Leary, 2008) and a history of alcohol-related convictions is associated with intimate partner violence perpetrated by handgun owners (Laqueur et al., 2019). We also found an association between firearm access in our sample and employment. Alcohol use disorder is a known risk factor for workplace violence (McFarlin et al., 2001), and co-workers are responsible for a significant portion of homicides involving firearms in the workplace (Doucette et al., 2019). Therefore, interventions to improve safe storage of guns by patients with substance use disorders undergoing medically supervised withdrawal and to educate them about the risks owning a firearm while grappling with a substance use disorder may reduce risk of harm not only to themselves, but also to their partners and coworkers.

We acknowledge that a primary limitation of this study is that our results are based on patient self-report and that patients may have withheld information about firearm access due to a variety of reasons including fears that their firearms might be confiscated and having legal histories that make possession of a firearm illegal. Another limitation is that individuals administering the screening instrument did not receive formal training on its delivery, so there are likely some inconsistencies in the manner in which they posed screening questions to patients and how they interpreted and coded particular answers provided by patients. There was also no formal evaluation of a patient's socioeconomic status and limited useful proxies exist outside of employment status, which was used in this study.

The factors that contribute to firearm ownership and access are complex (Pierre, 2019) and dynamic, and deserve further investigation in patients with substance use disorders. This study provides another example where standardized screening for firearm access greatly improves rates of completed documentation for this serious suicide and homicide risk factor among those with substance use disorders. Further research investigating the effectiveness of proposed clinical recommendations for firearm screening, and discussions about safe storage are needed (Roszko et al., 2016; Wintemute et al., 2016). To date, no formal clinical guidelines exist to support clinicians in these discussions.

## Contributors

Dr Jeremy Weleff, Dr Brian Barnett, Dr David Streem, Robert Butler.

## Role of funding source

Nothing declared. No funding.

## Disclosure statement

Dr. Barnett has received stock options from CB Therapeutics as compensation for advisory services. He also receives monetary compensation for editorial work for DynaMed Plus (EBSCO Industries, Inc). The other authors report no relations with commercial entities. This work was unfunded.

## Declaration of Competing Interest

No conflict declared. Dr. Barnett has received stock options from CB Therapeutics as compensation for advisory services. He also receives monetary compensation for editorial work for DynaMed Plus (EBSCO Industries, Inc). The other authors report no relations with commercial entities. This work was unfunded.
